# Focal vs. fecal: Seasonal variation in the diet of wild vervet monkeys from observational and DNA metabarcoding data

**DOI:** 10.1002/ece3.9358

**Published:** 2022-10-01

**Authors:** Loïc Brun, Judith Schneider, Eduard Mas Carrió, Pooja Dongre, Pierre Taberlet, van de Waal, Luca Fumagalli

**Affiliations:** ^1^ Laboratory for Conservation Biology, Department of Ecology and Evolution, Biophore University of Lausanne Lausanne Switzerland; ^2^ Department of Ecology and Evolution, Biophore University of Lausanne Lausanne Switzerland; ^3^ Inkawu Vervet Project Mawana Game Reserve, Swart Mfolozi KwaZulu Natal South Africa; ^4^ Laboratoire d'Ecologie Alpine Université Grenoble Alpes, CNRS Grenoble France; ^5^ UiT – The Arctic University of Norway, Tromsø Museum Tromsø Norway; ^6^ Swiss Human Institute of Forensic Taphonomy, University Centre of Legal Medicine Lausanne‐Geneva, Lausanne University Hospital and University of Lausanne Lausanne Switzerland

**Keywords:** diet estimation, DNA metabarcoding, environmental DNA, method comparison, primates, seasonal variation

## Abstract

Assessing the diet of wild animals reveals valuable information about their ecology and trophic relationships that may help elucidate dynamic interactions in ecosystems and forecast responses to environmental changes. Advances in molecular biology provide valuable research tools in this field. However, comparative empirical research is still required to highlight strengths and potential biases of different approaches. Therefore, this study compares environmental DNA and observational methods for the same study population and sampling duration. We employed DNA metabarcoding assays targeting plant and arthropod diet items in 823 fecal samples collected over 12 months in a wild population of an omnivorous primate, the vervet monkey (*Chlorocebus pygerythrus*). DNA metabarcoding data were subsequently compared to direct observations. We observed the same seasonal patterns of plant consumption with both methods; however, DNA metabarcoding showed considerably greater taxonomic coverage and resolution compared to observations, mostly due to the construction of a local plant DNA database. We found a strong effect of season on variation in plant consumption largely shaped by the dry and wet seasons. The seasonal effect on arthropod consumption was weaker, but feeding on arthropods was more frequent in spring and summer, showing overall that vervets adapt their diet according to available resources. The DNA metabarcoding assay outperformed also direct observations of arthropod consumption in both taxonomic coverage and resolution. Combining traditional techniques and DNA metabarcoding data can therefore not only provide enhanced assessments of complex diets and trophic interactions to the benefit of wildlife conservationists and managers but also opens new perspectives for behavioral ecologists studying whether diet variation in social species is induced by environmental differences or might reflect selective foraging behaviors.

## Graphical abstract

In this study, we compare observational and eDNA methodologies for studying plant and arthropod diet items of wild vervet monkeys (*Chlorocebus pygerythrus*). We observed the same seasonal patterns with both methods, however, DNA metabarcoding showed considerably greater taxonomic coverage and resolution compared to observations. The application of a DNA metabarcoding approach can be useful not only for conservation studies aimed at disentangling complex diets or reveal trophic interactions, but also opens new perspectives for behavioural ecologists studying social species in the wild.

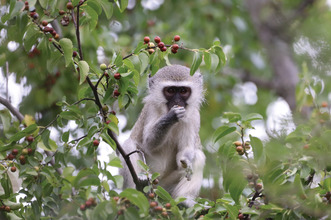

## 1 INTRODUCTION

Assessing a wild organism's diet is key to understanding its ecology and to highlight dynamics of communities and ecosystems through species' trophic interactions (Duffy et al., 2007). Traditionally employed methods, e.g. direct observations, microhistology of feces or gut contents, fatty acid, and stable isotope analysis, encounter certain limits when analyzing the diet of generalist and omnivorous species or attempting to disentangle the structure of complex food webs (Nielsen et al., 2018; Pompanon et al., 2012). The advent of DNA metabarcoding (Taberlet et al., 2012) and the simultaneous assessment of heterogeneous species mixes provide a valuable technique to open new perspectives in ecological network analysis (Clare, 2014). DNA metabarcoding studies using feces cover a range of different aims, such as diet characterization (Burgar et al., 2014; De Barba et al., 2014; Shehzad et al., 2012), parallel prey and predator identification (Galan et al., 2018; Gillet et al., 2015), or biodiversity assessment (Nørgaard et al., 2021; Shao et al., 2021). Some studies include different variables such as endoparasites and sex ratios along with the diet (Swift et al., 2018), or the predator's population structure (Bohmann et al., 2018). For many research questions in ecology, robust estimations of biomass or abundances are necessary for meaningful results going beyond simple detection or non‐detection (Pimm et al., 2014). Therefore, a number of studies show the method's potential for assessing complex correlations relying on its semi‐quantitative explanatory power when studying, for example, niche partitioning (Arrizabalaga‐Escudero et al., 2018; Kartzinel et al., 2015; Pansu et al., 2019; Vesterinen et al., 2018) or intrapopulation variation (Voelker et al., 2020).

In many cases, reliable abundance data can be obtained by observation; however, there is an ongoing debate about the quantification potential of eDNA‐based methods (Deagle et al., 2019; Zinger et al., 2019). For example, PCR primer‐induced biases, i.e. the preferential amplification of certain taxa and the under‐ or non‐representation of others, are considered a main source of biases in DNA metabarcoding (Jusino et al., 2019; Piñol et al., 2015; Piñol et al., 2019). Data treatment also influences the outcome (Calderón‐Sanou et al., 2019); occurrence data supposedly inflate rare taxa but are less sensitive to PCR‐introduced biases whereas the use of relative read abundances (RRA) may better account for variations in biomass (Deagle et al., 2019). RRA correspond to the number of reads of a sequence in a sample divided by the total number of reads of the same sample. Relative data do not only account for the presence of taxa in a sample but are expected to correlate to some extent with the amount of DNA present in the sample, therefore representing a semi‐quantitative approach. In this study, we used RRA data, maintaining identical experimental conditions for all samples to minimize biases and to allow for comparisons.

The DNA metabarcoding approach has been used only recently for diet studies in primatology, as the research field has traditionally relied on various observational methods for behavioral studies (but see Lyke et al., 2018; Mallott et al., 2017, 2018; Mallott et al., 2015; Osman et al., 2020; Quéméré et al., 2013; Rowe et al., 2021). Inter‐method comparisons are useful to test different methods' reliabilities and congruencies to assess consistency of results. However, the aim is not only to compare performances but also to determine under which circumstance the complementary use of these methods is advisable to allow their optimal application in future studies. Since in many cases observational feeding data are available, but with weak taxonomic resolution and/or with a limitation due to feeding habits that are difficult to observe, complementing these data by a DNA metabarcoding approach may be beneficial.

**FIGURE 1:**
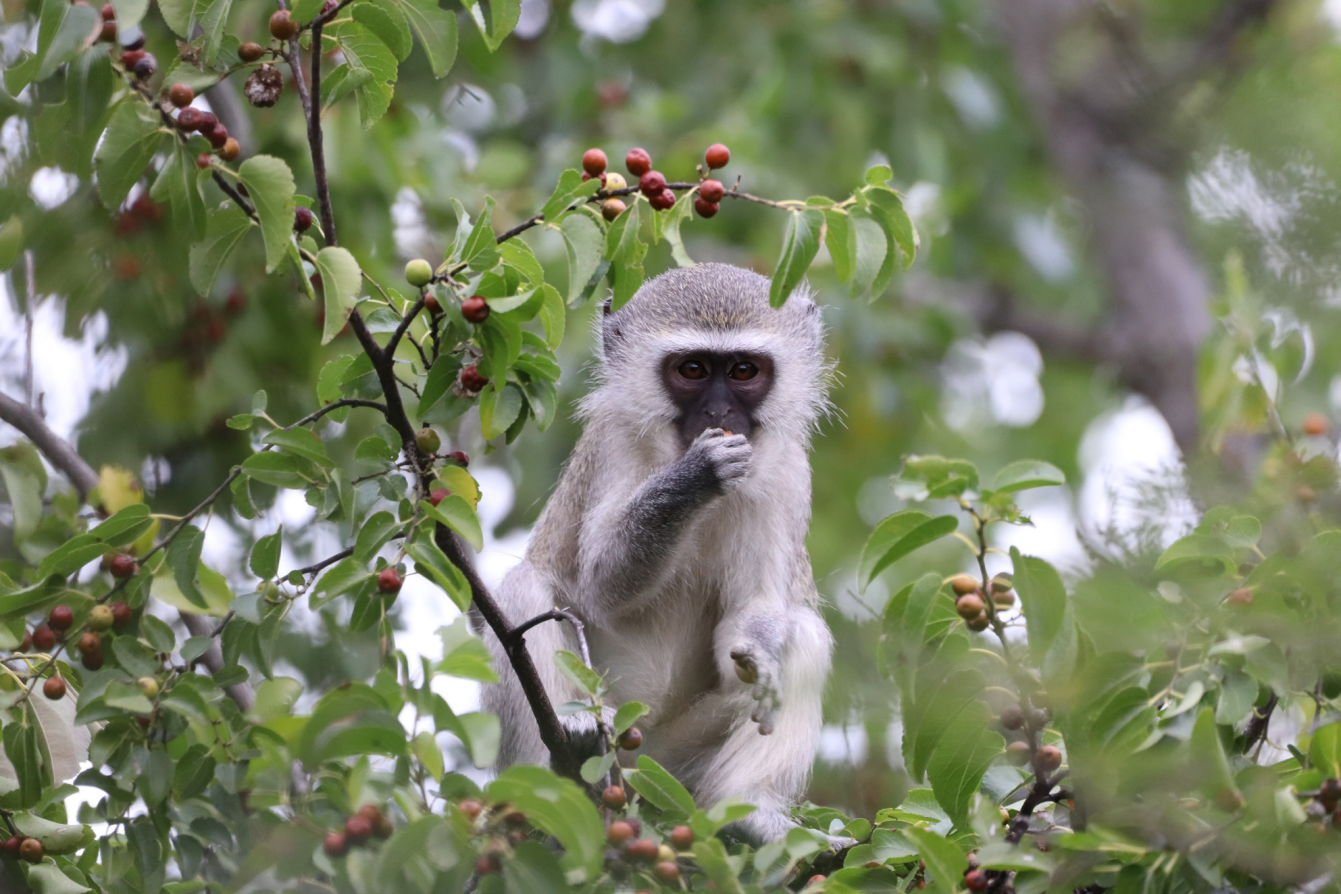
Juvenile vervet monkey (*Chlorocebus pygerythrus*) feeding on fruits of *Ziziphus mucronata*. © Michael Henshall.

To this aim, we compared dietary variation inferred from DNA metabarcoding to direct observations, in an opportunistic and generalist primate, the vervet monkey (*Chlorocebus pygerythrus*, Figure 1). Vervet monkeys are omnivorous and previous observational studies found that they feed mainly on trees, invertebrates, and occasionally small vertebrates (Barrett, 2009; Tournier et al., 2014). We analyzed 823 fecal samples of 130 individuals from four neighboring wild groups collected over 1 year, with two DNA metabarcoding assays targeting plant and arthropod components of the diet. The study of omnivorous species represents certain challenges (Tercel et al., 2021) that will be addressed in the discussion. The aim of the present study was threefold: (a) compare taxonomic coverage and resolution between observational and DNA metabarcoding data, (b) establish the most complete dietary profile in a wild vervet monkeys' population, and (c) assess resource use by vervet monkeys across seasons.

## 2 MATERIALS AND METHODS

### 2.1 Study site and subjects

The study was conducted between 09/2017 and 02/2019 as part of the Inkawu Vervet Project (IVP) in the Mawana game reserve (28°00.327S, 031°12.348E), KwaZulu Natal, South Africa. IVP was founded in 2010 and research has been conducted ever since on wild vervet monkeys mainly in the field of behavioral ecology, demonstrating the high social learning capacity of this species (Whiten & van de Waal, 2018). Our study includes four neighboring groups that are routinely followed by researchers. All individuals were identified using specific bodily and facial features (e.g. scars, colors, shape). The vegetation of the study site is classed as Savannah biome, characterized by areas of grasslands with dispersed singular or clusters of trees forming a mosaic with the typical savannah thornveld, bushveld, and thicket patches (Mucina & Rutherford, 2006). Each dataset, observational and DNA metabarcoding data, covered a period of 12 months, but they overlapped for 6 months only due to temporary constraints on focal sampling activities. Meteorological data assessed for the whole sampling period do not show major variation between the two sampled years for rainfall and temperature (Appendix S1: Figure S1). Therefore, we expected season to have a greater impact in terms of vegetation variation than the year of sampling and we consequently compared the data per month/season regardless of the year. Seasons were defined as follows, with the middle of a month as the seasonal delimitation (van Wyk & van Wyk, 2013): August–November (spring), November–March (summer), March–May (autumn), and May–August (winter).

### 2.2 Observational data

The observational data used for this study were obtained by instantaneous focal animal sampling methods on 101 adult group members between 09/2017 and 08/2018. In focal samplings, the focal individual is followed for a defined period and occurrences of (inter)actions are recorded, but parameters can vary according to specific study designs (Altmann, 1974). Here, each focal sample lasted 20 min and the focal animal's behavior was recorded instantaneously every 2 min resulting in 10 data points per focal sample (6176 focal screenings in total). Observers chose focal animals opportunistically, with the aim to collect one full focal sample per individual across three different time windows (morning, midday, afternoon), every 10 days. Total length of the data collection periods per day varied throughout the year according to sunrise and sunset times, while being equally distributed between the three daily time windows covering all daylight hours. Prior to data collection, all IVP observers had to pass an inter‐observer reliability test with a minimum Cohen's kappa value of 0.8 for each data category with an experienced researcher. Data were collected on tablets (Vodacom Smart Table 2, equipped with Pendragon Forms version 8). From the complete dataset, we extracted all feeding observations and created separate datasets for plant and arthropod items. The focal dataset for plants contained 19,406 observations, of which 12,315 identified plant genera or species (63.46%). The arthropod dataset contained 1359 observations (of which 15.82% indicated broad insect categories, i.e. termites or grasshoppers). Plant and arthropod observations that only occurred once were omitted from the final dataset.

### 2.3 Local plant database

In the field, 54 plant species were morphologically identified and collected (van Wyk & van Wyk, 2013). These include all species confirmed by previous observation of feeding behavior in the area and other frequently occurring plants that could potentially be consumed. Sampled material from each species was stored in silica gel until DNA extraction using the DNeasy Plant Mini Kit (Qiagen) with a final elution in 100 μl AE buffer. To construct a local database, the whole chloroplast *trn*L (UAA) intron, which comprises the P6 loop targeted in the DNA metabarcoding assay described below, was amplified with primers c/d (Taberlet et al., 2007). The PCR reactions were performed in 25 μl. The mixture contained 1× PCR Gold Buffer (Thermo Fisher Scientific), 2 mM MgCl_2_, 0.2 mM of dNTPs, 0.04 μg of bovine serum albumin (Roche Diagnostics), 0.5 μM of forward and reverse primers, 1 U of AmpliTaq Gold (Thermo Fischer Scientific), and 2 μl of template DNA. PCR cycling conditions were 10 min denaturation at 95°C, followed by 35 cycles of 30 s at 95°C, 30 s at 50°C, and 1 min at 72°C, with a final elongation step of 5 min at 72°C. PCR products were purified using the QIAquick PCR Purification Kit (Qiagen) before Sanger sequencing in both directions at Microsynth AG. The obtained P6 loop sequences were used for our reference database. The final database consisted of 48 sequences matching 54 species (i.e. 43 unique sequences, four sequences shared between two species and one sequence shared between three species, Appendix S1).

### 2.4 Fecal sample collection

A total of 823 fecal samples of 130 known individuals were collected during a 12‐month period (03/2018 to 02/2019, Figure 2). Whenever a specific individual was observed defecating, the inner part of the scat was immediately collected unless it had already been sampled the same day or if an experiment involving food rewards had been conducted with the group in the 48 preceding hours. Approximately 0.5 cm^3^ was collected with gloves and a disposable plastic spoon from inside the scat into 20 ml HDPE scintillation vials (Carl Roth GmbH) and covered with 10 ml absolute ethanol. After 24–36 h, the ethanol was replaced by silica gel beads and samples stored until DNA extraction.

**FIGURE 2:**
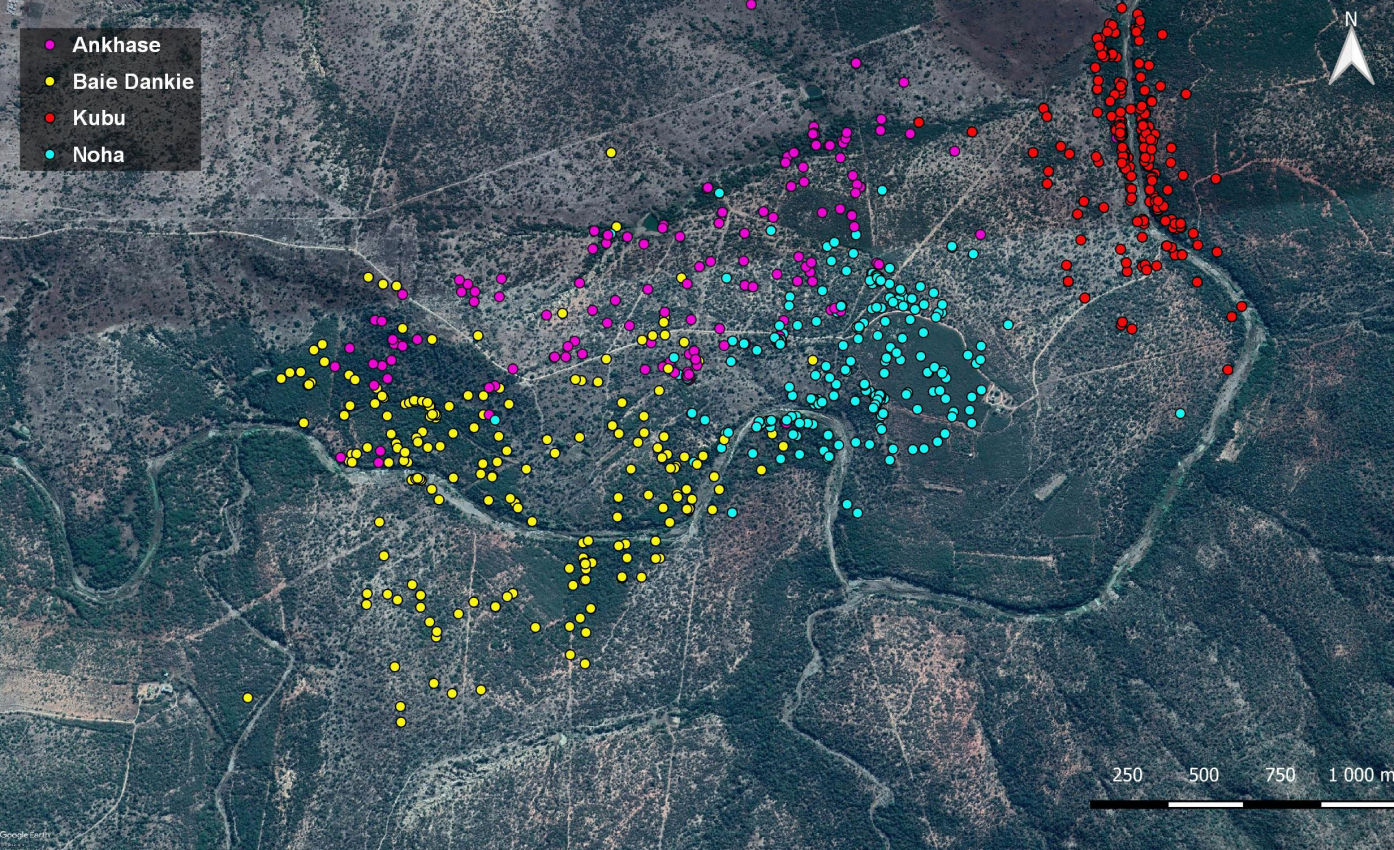
The map indicates the sampling locations of the 823 fecal samples of 130 individuals in the Inkawu Vervet Project, South Africa. The different groups are represented by different colored dots: Ankhase: purple (*n* = 146), Baie Dankie: yellow (*n* = 212), Kubu: red (*n* = 224), Noha: blue (*n* = 241).

### 2.5 DNA metabarcoding

#### 2.5.1 DNA extraction

DNA extraction of scat samples was performed using a phosphate buffer‐based approach (Taberlet et al., 2018) following a modified protocol of the NucleoSpin Soil Kit (Macherey‐Nagel). Scats were directly transferred from the scintillation vials into 2 ml Eppendorf tubes with 1.3 ml of saturated phosphate buffer. For a better absorption of the DNA, the samples were homogenized by vortexing before spinning on a tube rotator for 15 min. The suite of the protocol was as recommended using the QIAvac technology (Qiagen), with a final elution in 100 μl of SE buffer. Extractions were performed in a pre‐PCR laboratory exclusively dedicated to low DNA‐content analyses (Laboratory for Conservation Biology, University of Lausanne). A subset of the extractions was tested for inhibitors with real‐time quantitative PCR (qPCR) applying different dilutions in triplicates. qPCR reagents and conditions were the same as in DNA metabarcoding PCR (see below), but for 45 cycles and with the addition of SybrGreen (Thermo Fischer Scientific). Following these analyses, all samples were diluted 5‐fold.

#### 2.5.2 DNA metabarcoding assay

DNA extracts were amplified in triplicates with two sets of primers. The first one targets the plant components of the diet amplifying the P6 loop of the *trn*L intron (UAA) of chloroplast DNA (10–220 bp, Sper01 (Taberlet et al., 2018) corresponding to g/h (Taberlet et al., 2007)). The second primer pair amplifies a fragment of 16S mitochondrial rDNA within the phylum Arthropoda (76–168 bp, Arth02 (Taberlet et al., 2018)). For the latter, one blocking oligonucleotide (5′‐AGGGATAACAGCGCAATYCTATTCTAGAGTC‐C3‐3′) was added, designed specifically for this study to limit the amplification of both human and vervet monkey DNA (for specifications see Appendix S1: Figure S2 and Taberlet et al., 2018). PCR reactions were performed in a final volume of 20 μl in 384‐well plates. The mixture contained 1 U AmpliTaq Gold 360 mix (Thermo Fischer Scientific), 0.04 μg of bovine serum albumin (Roche Diagnostics), 2 μM of human‐blocking primer (coupled with Arth02 primers only), 0.2 μM of tagged forward and reverse primers (i.e. primers with eight variable nucleotides added to their 5′‐end, allowing sample identification), and 2 μl of template DNA. PCR cycling conditions were 10 min at 95°C, followed by 40 cycles of 30 s at 95°C, 30 s at 49 or 52°C for Arth02 and Sper01, respectively, and 1 min at 72°C, with a final elongation step of 7 min at 72°C. For each assay, extraction negative, PCR negative (H_2_O), and positive controls as well as blanks were included. The positive controls of DNA mixtures of known concentrations were added in order to control for amplification success and were composed of species not expected in the study site (Appendix S1: Table S2), sequences were added to the respective databases. The inclusion of blanks, i.e. completely empty wells, allows to detect artifactual sequences after tag switches during the sequencing process (Schnell et al., 2015). Amplification success was verified for a subset of samples, using the QIAxel technology (Qiagen). All PCRs were performed at the Laboratoire d'Ecologie Alpine (LECA).

PCR reactions were pooled per replicate before library preparation, i.e. resulting in six separate libraries (i.e. three per metabarcode) each containing 823 samples plus controls. Amplicon pools were purified using the MinElute PCR Purification Kit (Qiagen) and quantified using a Qubit 2.0 Fluorometer (Life Technology Corporation). Library preparation was performed using the TruSeq DNA PCR‐Free Library Prep Kit (Illumina) starting at the repair ends and library size selection step with an adjusted beads ratio of 1.8 to remove small fragments. After adapter ligation, libraries were validated on a fragment analyzer (Advanced Analytical Technologies). Final libraries were quantified by qPCR, normalized and pooled before 150 paired‐end sequencing on the Illumina Miniseq Sequencing System with a High‐Output Kit, yielding up to 25 million reads (Illumina).

#### 2.5.3 Bioinformatic data treatment

Bioinformatic processing of raw sequences was conducted separately for each library using the *OBITools* package (Boyer et al., 2016). Forward and reverse reads were assembled with a minimum quality score of 40 and assigned to samples based on unique tag and primer combinations, allowing two mismatches with primer, and identical sequences were clustered. All sequences with <10 reads per library were discarded as well as those not corresponding to primer specific barcode lengths, i.e., 10–220 bp for Sper01 and 76–168 bp for Arth02 (Taberlet et al., 2018). This was followed by two different clustering methods. First, pairwise dissimilarities between reads were computed and lesser abundant sequences were clustered into the most abundant ones. Second, we reduced remaining clusters based on a sequence similarity of 97% using the *sumaclust* algorithm (Mercier et al., 2013). For taxonomic assignment of sequences, three different reference databases were used. The *local* database for Sper01 was based on the local plant collection (see 2.3). Furthermore, to construct *global* databases, both primer sets were used to simulate in silico PCRs on GenBank using the *ecoPCR* software (Ficetola et al., 2010) to select all sequences corresponding to our primers (restrained to three mismatches, the targeted barcode lengths and to Metazoa and Viridiplantae, respectively). Sper01 sequences were first assigned to the local database and non‐assigned sequences were subsequently run against the global Sper01 database, both with 97% thresholds. In addition, in order to test the effect of the local database, we did the taxonomic assignment of Sper01 sequences with only the global database and assessed the ratio of assigned sequences. Arthropod sequences were directly run against the global Arth02 database with a 97% similarity threshold.

Additional filtering of sequences and subsequent data analyses were performed in R (version 4.0.2; R Core Team, 2022). Sequences that were more abundant in extraction and PCR controls than in samples were considered as contaminants and removed. To account for tag switching, we considered the leaking of a sequence to be directly linked to its abundance. To test this, we performed Wilcoxon signed‐rank tests to assess the relationship between samples and blanks and a ratio was defined independently for each library to remove likely leaked sequences, as implemented in the R package *metabar* (Zinger et al., 2021). Replicates per sample were compared and the mean number of reads was retained if a sequence was present in at least two out of three replicates, in line with Ficetola et al. (2015) and a minimum of five reads. All plant species‐level assignments were manually verified and re‐assigned to genus level if the known geographic species range did not match but the genus was known to occur in South Africa, else to family. For Arth02, we retained only the family level to avoid any taxonomic ambiguities (Meiklejohn et al., 2019) and all sequences assigned to vervets and humans were discarded.

### 2.6 Data analyses

Analyses on the sequence data were conducted using RRA if not stated otherwise. In order to treat the observational data similarly, the sum of observations of each consumed item per day was divided by the total number of focal screenings conducted that day. Sample numbers varied between months/seasons and methods, hence for subsequent analyses mean values were taken per temporal unit. Since data were not normally distributed (according to Shapiro–Wilk's tests), we employed non‐parametric tests. The impact of seasons on dietary variation was determined by principal coordinates analyses (PCoA) using the ade4 package (Dray & Dufour, 2007). To account for pseudo‐replication, the same weight was given here to all individuals, i.e. replicate samples sum up to 1 per specific individual, while observational data were aggregated per focal individual/season and transformed to relative abundances. We identified plant indicators for seasons using *Indicator value analyses* (Indval; Dufrêne & Legendre, 1997). Shannon–Wiener diversity indices were calculated per season (genera/species for plants, family level for arthropods) and Hutcheson *t* tests performed to test for significant differences between seasons (Hutcheson, 1970). We performed Mantel's tests (Mantel, 1967) implemented in the *vegan* package with 9999 permutations to compare the correlation between datasets with data aggregated per month and transformed to Bray–Curtis dissimilarity matrices. Spearman rank correlations were calculated for all plant species present in both datasets and with a minimum count of 350 in the focal dataset (with the exception of *V. nilotica*/*C. decapetala* and *E. crispa*/*E. undulata*/*D. dichrophylla* since sequence data matched two different species in the focal dataset).

## 3 RESULTS

The final dataset for Sper01 contained 5,275,361 reads assigned to 22 orders, 43 families, 61 genera, and 35 species. Of these 4,599,838 reads were assigned to 31 items with the local database, including 25 identifications at species level. Most of the plant genera and species consumed by this species are not only trees and shrubs but also cactuses, herbs, and grasses (Appendix S1: Table S3). Taxonomic assignment with solely the global database resulted in 330,612 reads assigned to 15 different species; however, only 10 species were reliable (Appendix S1: Figure S3). The taxonomic resolution was hence greatly increased with the local database allowing for more detailed analyses.

**FIGURE 3:**
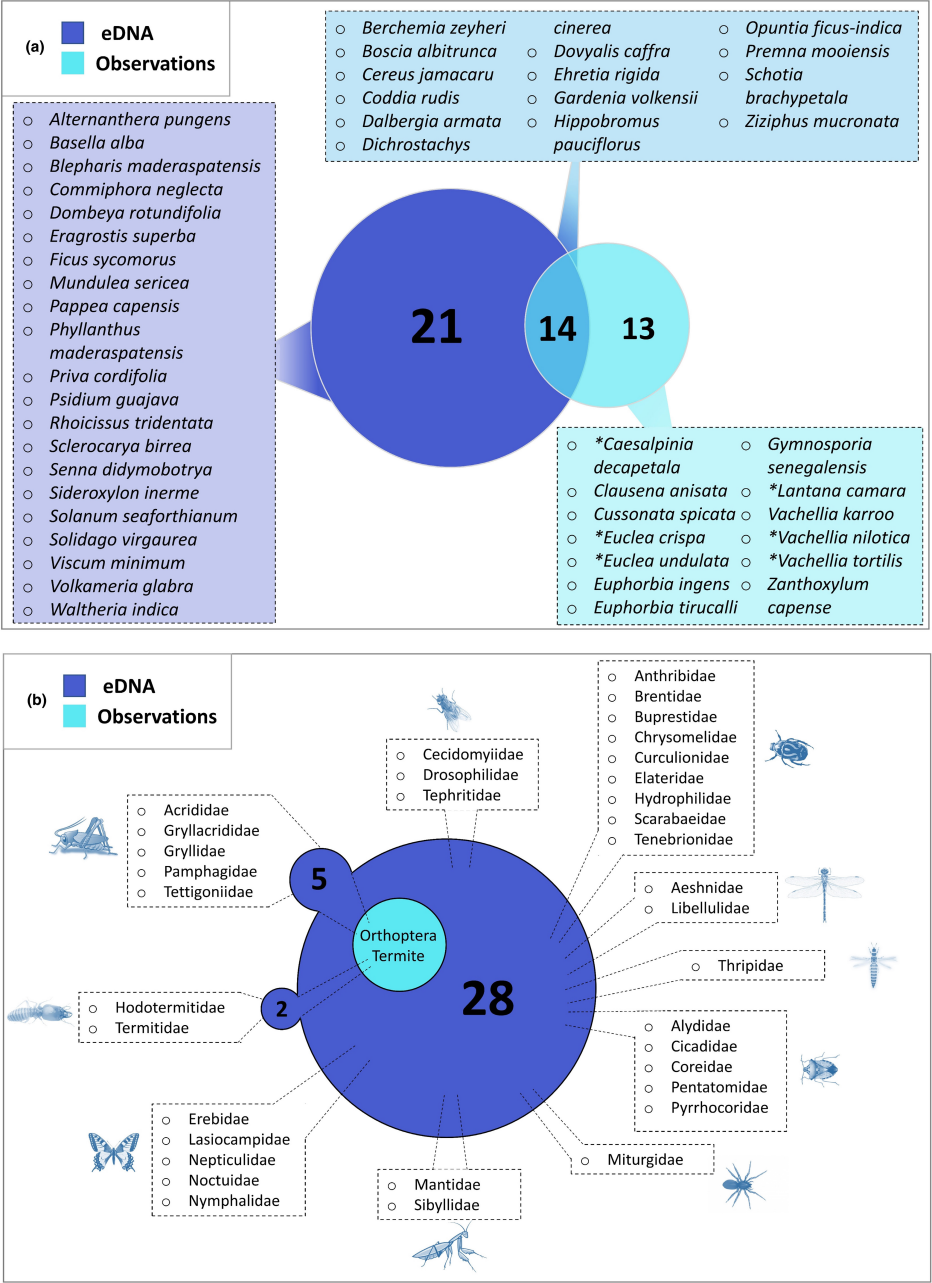
Venn diagrams. (a) Between consumed plant items at the taxonomic level of species detected by observation and eDNA. Plant species beginning with an asterisk (*) correspond to species for which the sequences amplified by the Sper01 metabarcode were identical between species as shown in Table S1. (b) Between arthropods detected by observation and eDNA. For eDNA data, the family level is included, whereas observations were limited to the order level for orthopterans and the infraorder level for termites. The two bubbles on the left side of the diagram indicate the families detected by eDNA that compose these two taxonomic groups. The category “undetermined insects” is not included for observations (see text). Rectangles separate the different orders illustrated by icons.

**FIGURE 4:**
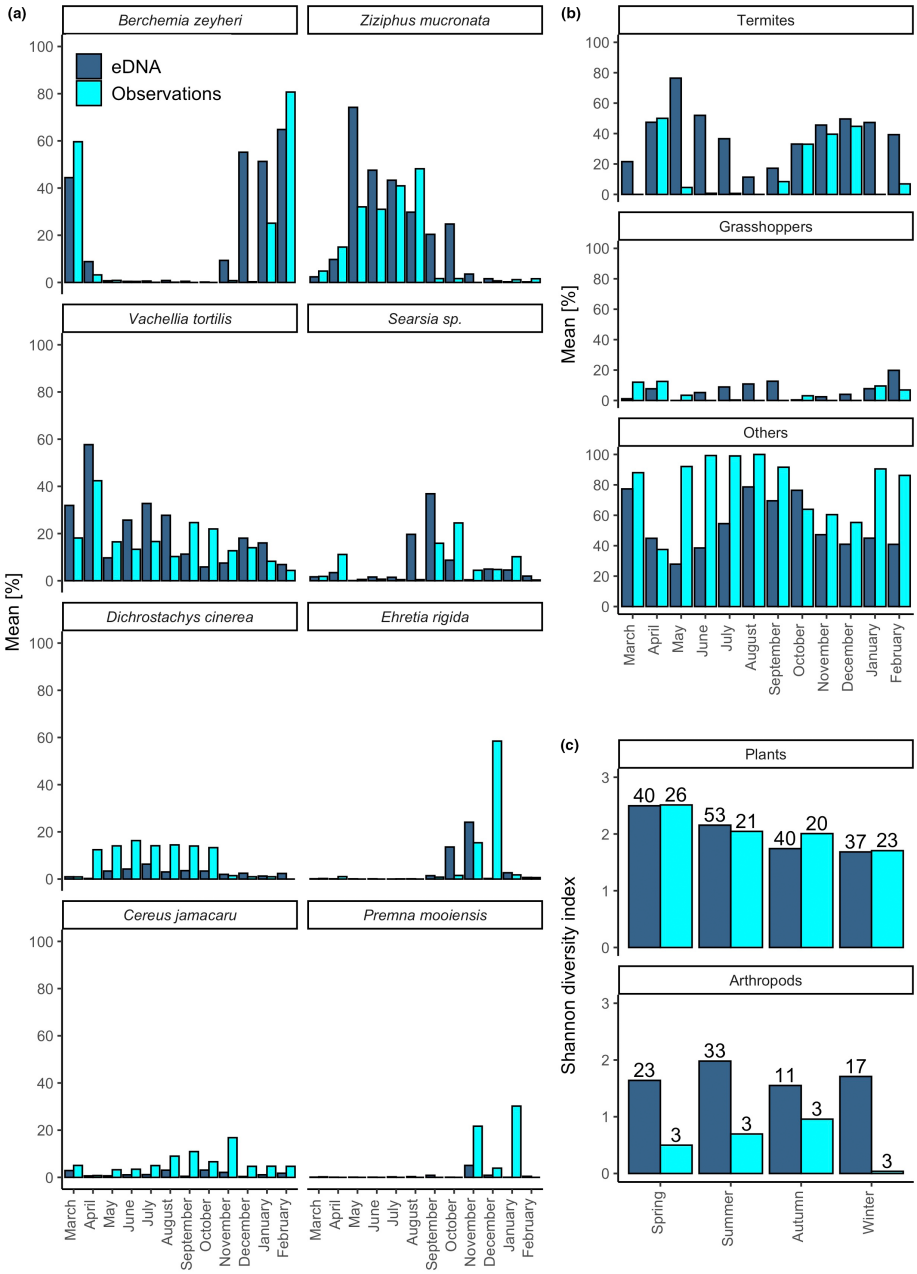
(a) Monthly comparison of DNA metabarcoding and observational data for the most frequent species in the focal dataset (>350 observations), with the exception of those that had identical metabarcodes and matched several species in the focal dataset. The observed plant *V. tortilis* corresponds to *V. tortilis/sieberiana* in the DNA metabarcoding dataset. Metabarcoding data are represented by the mean RRA and observational data by the mean count, both in percent. (b) Monthly comparison of DNA metabarcoding and observational data for “termites” (RRA of Hodotermitidae and Termitidae combined), “grasshoppers” (RRA of all detected families belonging to the order Orthoptera), and “others” (RRA of all remaining items). Metabarcoding data are represented by the mean RRA and observational data by the mean count, both in percent. (c) Shannon diversity index per season for observations and eDNA. There was no significant difference in diversity between methods (Hutcheson *t* test). Numbers on the bars indicate numbers of different observed/detected items per season. For plants, the included items are all observed/detected species and genera. For arthropods, the Shannon diversity was measured at family level for the metabarcoding data and for observational data based on the three categories (b).

**FIGURE 5:**
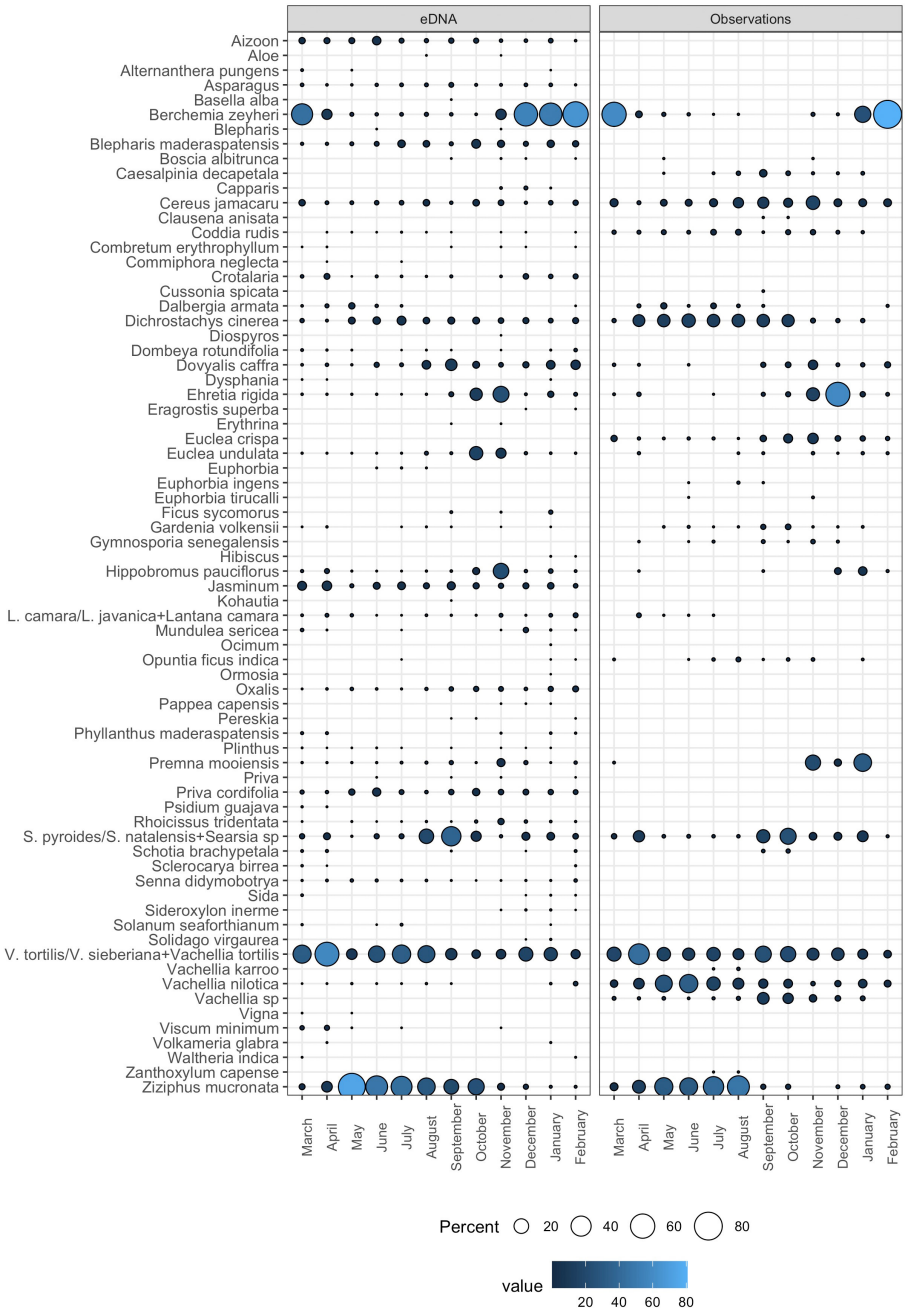
Mean RRA of plants genera and species in fecal samples per month (left) and mean of observations in focal follows per month (right). Note that the obtained sequence for Euphorbia is different from *E. ingens* and *E. tirucalli*. Also, *E. crispa* and *E. undulata* were identified to species level in the field but have identical sequences, the same is true for *V. nilotica* and *C. decapetala*; therefore, both entries for observations were kept but only one for eDNA. Several names in one line indicate identical sequences as well (on the left), but only one observed genus/species (on the right).

**FIGURE 6:**
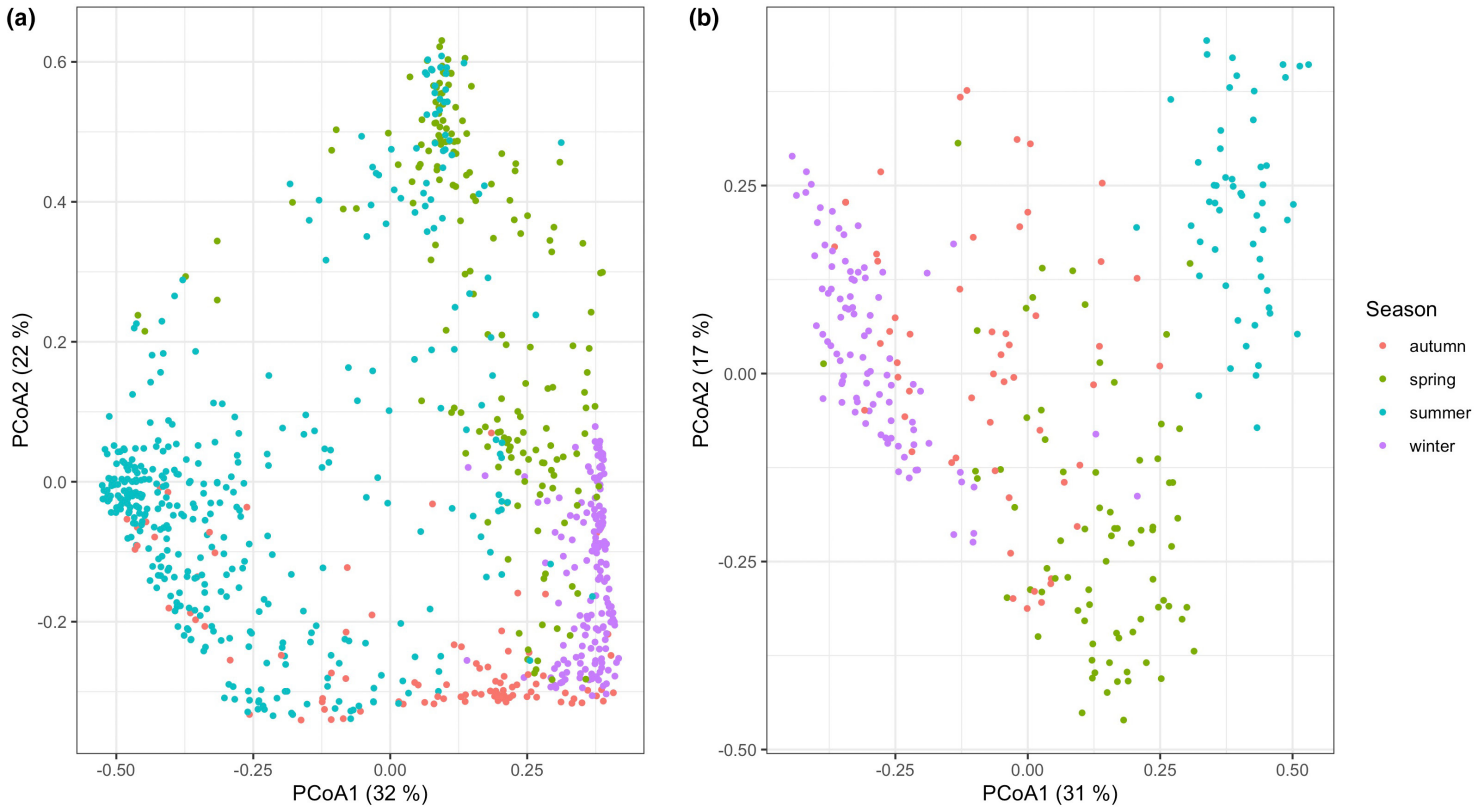
Principal coordinates analyses (PCoA) based on (a) relative read abundances (RRA) of consumed plants detected in fecal samples (*n* = 823) and (b) observational plant data of focal follow transformed to relative abundances per individual/season (*n* = 279). In brackets the relative Eigenvalues in percent.

During focal follows, vervet monkeys were observed feeding on 27 different plant species and two plant genera. Mean observations per month of the eight most frequent plant species in the focal dataset showed similar temporal patterns as the DNA metabarcoding data (Figure 4a) and a Mantel's test of Bray–Curtis dissimilarity matrices of data aggregated per month indicated a high correlation between methods (*r* = .62, *p* = 1e‐04). There was no positive correlation between methods for numbers of different diet items detected/observed per month (Appendix S1: Figure S4). However, positive Spearman rank correlations were observed when comparing single plant species, among which the most consumed ones (Appendix S1: Figure S5). In addition to the plant genera and species that were identified by both methods, DNA metabarcoding revealed 41 supplementary dietary items at this taxonomic level of which 21 at species level (Figures 3a and 5, Appendix S1: Table S3). The Shannon diversity did not differ significantly between both methods for plant genera and species observations/detections (Hutcheson t‐tests not significant) despite the variable total numbers, i.e. richness (Figure 4c). Seasonal shifts were most pronounced between the wet and the dry season for *B. zeyheri* and *Z. mucronata* indicating that one substitutes the other as principal food resource (Figure 4a). Season explained a lot of the variation in both datasets as illustrated by PCoAs (Figure 6a,b) and confirmed by *ANOSIM* with *R* = .51 and *R* = .57, both *p* = 1e‐04, for eDNA and observational data, respectively. Figure S6 shows observations and RRA over 12 months for seven plant species that were season indicators based on observational data. All except one, *C. jamacaru*, were indicator species in the metabarcoding dataset as well. The latter revealed several additional season indicator species (Appendix S1: Table S3).

**FIGURE 7:**
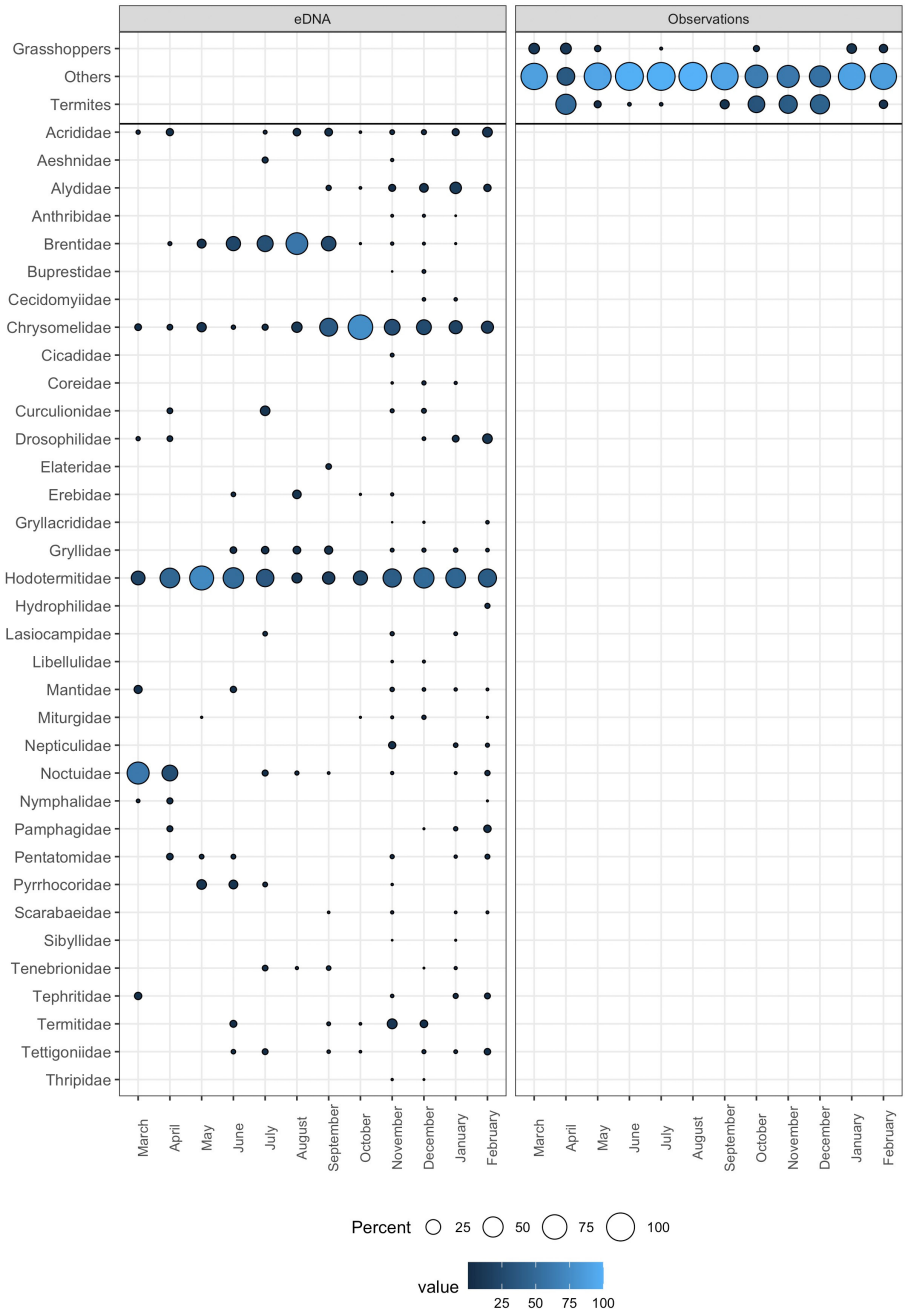
Mean RRA of arthropod families in fecal samples per month (left) and mean of observations in focal follows per month (right). The category “others” includes all insect observations that were neither identified as grasshoppers nor as termites. The families in the order Orthoptera (“grasshoppers”) are Acrididae, Gryllacrididae, Gryllidae, Pamphagidae, and Tettigoniidae. The families in the order Blattodea (equivalent to “termites”) are Hodotermitidae and Termitidae.

Over 12 months of observational focal sampling, there were in total 1359 foraging events for arthropods (1142 undetermined insects, 191 termites, 24 grasshoppers; Figure 3b). We investigated in particular the temporal dimension of the “termites” category since vervets feed on termites extensively during swarming periods, which can be easily observed. Figure 4b shows percentages of the occurrences of these categories together with the combined RRA data for the families Hodotermitidae and Termitidae (“termites”) as well as all taxa of the order Orthoptera (“grasshoppers”), and all other sequences combined (“others”). While a consistent trend was observed between methods, observations and DNA sequence data are not significantly correlated (Appendix S1: Figure S7).

Without relying on a reference database for taxonomic assignments, the Arth02 assay resulted in 1,698,439 sequences in total whereof, however, 961,542 belonged to vervets, leaving 736,897 reads clustered to 404 presumed arthropod operational taxonomic units (OTUs) (Appendix S1: Figure S3). By relying on the global database, the number of DNA sequences after final data filtering was 360,040 assigned to 11 orders and 35 families (Appendix S1: Table S4), i.e. 48.86% of reads were taxonomically assigned (not considering those of *C. pygerythrus*). The most abundant arthropod orders in terms of read counts and frequencies were Coleoptera, Blattodea, and Lepidoptera. We detected arthropod sequences in 96% of the samples in spring, 89.15% in summer, 58.59% in autumn, and 82.72% in winter, whereas the highest number of different orders and families was detected in summer, also showing the highest Shannon diversity (Figure 4c). While we observed monthly variation for certain taxa (Figure 7), there was overall a significant yet weak seasonal effect (Appendix S1: Figure S8).

## 4 DISCUSSION

The present study of vervet monkeys' diet over a 12‐month period highlights strong seasonal variation in consumed plants and less pronounced variation in arthropod consumption across seasons. The comparison of DNA metabarcoding data of plant diet components with field observational data shows similar patterns, in particular regarding relative abundances and seasonal variation. However, whilst observations captured well the main plant diet components, DNA sequencing data showed improved taxonomic coverage and resolution. With respect to arthropod consumption, DNA metabarcoding outperformed observations, allowing for a considerable expansion of the range of dietary items identified and demonstrating the clear advantages of this method to describe cryptic feeding behavior. Both methods have certain advantages and shortcomings as further discussed below, and genetic data are increasingly merged for network analyses with data from different sources to be used in a complementary way. For example, observational data provide in many cases more information regarding state and life stage of consumed items. While this may lead to more complete datasets, it implies also specific challenges as discussed by Cuff et al. (2022).

For plants items, our DNA metabarcoding assay detected many additional species and genera that had not been observed or identified to this level, as well as most of the species observed during focal follows. The taxonomic resolution was excellent for the plant assay due to the use of the local database (see also Quéméré et al., 2013). The increased detection by metabarcoding is likely due to observational difficulties in recording certain food items that are hard to identify or to observe, e.g. taking place in inaccessible or dense terrain (Matthews et al., 2020; Su & Lee, 2001). In our study, DNA metabarcoding further revealed consumption of otherwise well‐documented species in periods when they were missed during observations, likely due to the consumption of less visible parts, e.g. tree sap, or dried seeds or fruits collected from the ground.

All new information made available by DNA metabarcoding could imply important trophic relations that have been overlooked so far. This is particularly relevant for arthropod items, a food type rich in proteins and lipids (Rothman et al., 2014), for which feeding habits are poorly studied in primatology. Previous observational studies indicate feeding of vervets on arthropods with varying degrees of precision (Barrett, 2005; Struhsaker, 1967; Tournier et al., 2014) but detailed records have so far been missing. Here, with DNA metabarcoding, 35 different families representing 11 orders were identified and demonstrate increased diversity of arthropod consumption in vervets' diets compared to the three broad taxonomic categories grouping termites, orthopterans, and others as identified with observations (Figures 3b and 7 and Appendix S1: Table S4). For arthropods, dietary diversity and richness are hence markedly higher when relying on DNA metabarcoding (Figure 4c). Accordingly, we found no correlation between observational and genetic data (Appendix S1: Figure S7), indicating the aptitude of the latter to unmask new trophic interactions and to shed light on cryptic feeding behavior. A good example illustrated by our dataset is that of the twice‐yearly termite swarming, a major ecological event in South‐Africa (Lesnik, 2014), which was adequately captured by both our methods (Figure 4b). Although showing a similar trend, the observations and DNA sequence data are not significantly correlated. During swarming, the large number of flying termites emerging from the nest makes them highly visible to observers. However, during the rest of the year, when monkeys forage directly on the ground or in dead wood and in lesser quantities, most of these foraging events are cryptic or difficult to identify and thus missed by observers but documented by genetics. In general, observation of feeding on arthropods is particularly challenging (Pickett et al., 2012) and this is the likely cause of the minimal detail available from our observational data and previous observational studies on vervets. A comparison between observations and DNA metabarcoding yielded similar results for white‐faced capuchins (*Cebus capucinus*), with eight arthropod orders observed against 29 orders detected (Mallott et al., 2017). Furthermore, recent genetic studies on other primate species have similarly contributed to a better representation of arthropod diet components, either by using a cloning approach (Pickett et al., 2012), DNA metabarcoding (Lyke et al., 2018; Mallott et al., 2017, 2015; Rowe et al., 2021), or metagenomic sequencing (Srivathsan et al., 2015). This study demonstrates the advantages of using DNA metabarcoding alongside observations, adding to previous findings for the part of plants and arthropods of the diet of wild vervets.

In line with previous work showing that movements of vervets were mostly driven by plant resource availability, and therefore seasonality (Barrett, 2009), we found significant variation in plant consumption, largely shaped by the dry and wet seasons (Figure 6). For the plant genera and plant species that have been recorded with both methods, we found comparable abundances, similar seasonal patterns, and season indicator species (Appendix S1: Figure S6, Table S3). Our inter‐method comparison illustrates for certain plant species very clear temporal correlations (Figure 4a, Appendix S1: Figure S5). Regarding plants, both methods indicated similar Shannon indices per season but the genetic approach resulted in higher dietary richness (Figure 4c). While some plants are consumed continuously (different parts may be eaten over the year), the consumption of others was associated with particular seasons (e.g. strong association of *Z. mucronata* with winter). Previous studies on vervets found that they spend more time foraging in the dry season because of resource scarcity (Arseneau‐Robar et al., 2017; Canteloup et al., 2019). They can hence be expected to be more opportunistic feeders in the dry season than when food is abundant in the wet season and the opportunity to engage in selective foraging behaviors arises. During wet, food‐abundant summer, we detected a higher diversity of consumed items in the scat samples. This shows that vervets adapt their diet according to available resources.

Concerning arthropod consumption, although the statistical effect of season on arthropod consumption was weak, the highest percentage of samples containing arthropod sequences was found in spring and summer, as well as the highest (family) richness and Shannon diversity (Figure 4c). Given the very different numbers of arthropod items detected per method, the comparable diversity might surprise but can be explained by the dominance of few abundant families/categories; this may be different in other study contexts. Overall, our results show that season is an important variable for diet choice; therefore, sampling designs should take it into account when this is relevant for the research question. Here, selective behaviors are most likely in the wet season when differences are the most accentuated and resources are not limiting, hence future sampling could focus on that season to capture most efficiently any behavioral differences that are not driven by resource availability, as discussed below.

DNA metabarcoding approaches do nonetheless entail their own limitations, some are marker specific and some are methodological. Primer‐induced biases may have led to under‐ or non‐representation of certain arthropod taxa in this study. The study of omnivorous species is often neglected and thus highly necessary but requires in most cases the combination of different primer sets, which increases study cost and introduces new challenges (Tercel et al., 2021). Plants and arthropods were considered the most important targets based on observational data; however, our marker choice excluded the detection of other dietary items (i.e. feeding on birds, eggs, and mushrooms was occasionally observed). Some plant species shared identical sequences in the metabarcode we amplified, making it impossible to differentiate genetically between them (Taberlet et al., 2007). For plants observed only in small numbers and not detected (false negatives), this may be due to stochastic reasons and the fact that observations and scat samplings were not conducted at the same time. For the observed but not detected *V. karroo* and *Z. capense* there is no sequence available in our databases. While this can be overcome by including further sequences, it points to the issue of incomplete databases in metabarcoding studies (Furlan et al., 2020; Taberlet et al., 2012). A local database would certainly increase the taxonomic coverage and resolution as well for the Arth02 assay and would have allowed the attribution of some abundantly represented OTUs, in particular since our research is pursued in a geographic region underrepresented in genetic databases (Kvist, 2013; Marques et al., 2021). In addition, unlike observational data, genetic data cannot detail which part and state of the plant or which life stage of an arthropod has been consumed (Pompanon et al., 2012; Rees et al., 2014). Parts of the sequences may be due to secondary ingestion, accidental consumption, or of parasitic origin and not represent (intentionally) consumed items (Tercel et al., 2021); therefore, interdisciplinary studies with parasitology may be fruitful. Arthropods may have ingested plant DNA that we thus falsely detected as part of vervet diet, and at the other end of the spectrum unintentional feeding of arthropods is possible, e.g. of small Thripidae. The feeding on termites and grasshoppers is confirmed by observations, and also active foraging (i.e. vervets searching for insects), showing once more the benefit of complementary use of methods.

Choices made during the processing of DNA metabarcoding data may influence the outcome of these studies (Calderón‐Sanou et al., 2019). In this study, we applied a stringent filtering of the data to avoid spurious DNA, using percentual and absolute thresholds. It has been argued that arbitrary minimum copy thresholds might omit true sequences (Littleford‐Colquhoun et al., 2022) and that percentual thresholds were more suitable in case of uneven sequencing depths (Drake et al., 2022). To avoid the generation of supplementary biases, it is recommended to normalize PCR amplicons before pooling. Here we accepted the risk of missing some true detections by omitting items with very small read counts, which may also affect samples with uneven sequencing depths differently. Another point is the transformation of read counts; while most studies traditionally rely on occurrence data, others argue that RRA data might better capture ecological signals (Deagle et al., 2019; Kartzinel et al., 2015; Voelker et al., 2020). Here, we chose RRA and although it may entail biases, the comparison to observational data validates this choice. For example, two of the most consumed plants throughout the year, *B. zeyheri* and *Z. mucronata*, represent very variable proportions of the diet depending on the season. Categorical data would not show any variation here; however, we observed strong seasonal patterns with both RRA and observational data (Figure S6). A recent diet study targeting the same genetic region found positive correlations between the RRA of plant families in fecal samples and the observed duration spent feeding on those (Mallott et al., 2018).

The taxonomic coverage and resolution as well as the methodological standardization (including no inter‐observer variability) point to the benefits of environmental DNA (eDNA)‐based surveys. Depending on the species studied, DNA metabarcoding represents cost‐ and labor‐effective alternatives or complements to traditional methods (Mena et al., 2021) and sequencing costs are likely to further decrease in the near future. The sensitivity, taxonomic resolution, and non‐invasiveness of the method are major advantages in conservation research (Thomsen & Willerslev, 2015). There is great potential to learn more about, for example, nocturnal, arboreal, and other elusive species and/or the adaptive potential of fragmented populations (Quéméré et al., 2013). Many primates are threatened and of high conservation concern (IUCN, 2020; Schwitzer et al., 2017). There is thus a need for robust data to inform empirically based conservation strategies (Pimm et al., 2014), where diet studies are undoubtedly of primary interest. Although it remains challenging to properly assess to what extent the final data represent the biomass of food items initially ingested, controls incorporated throughout the study and appropriate knowledge of the ecology enable valuable insights going beyond traditional approaches. DNA metabarcoding has thus great potential to bring new insights on foraging behaviors and ultimately, on the underlying mechanisms shaping such behaviors.

Our study demonstrates benefits of an interdisciplinary approach. Moreover, this study being the first validating the use of eDNA to assess diet in our system, future analyses may investigate whether variation in individual or group diet is induced by environmental differences or if it might reflect selective foraging behaviors. Therefore, the application of a DNA metabarcoding approach can be useful not only for conservation studies aimed at disentangling complex diets or reveal trophic interactions but also opens new perspectives for behavioral ecologists and cultural evolutionists studying social species in the wild.

## AUTHOR CONTRIBUTIONS

**Loic Brun:** Formal analysis (equal); investigation (equal); writing – original draft (equal). **Judith Schneider:** Formal analysis (equal); investigation (equal); writing – original draft (equal). **Eduard Mas‐Carrió:** Investigation (equal); writing – review and editing (equal). **Pooja Dongre:** Investigation (equal); writing – review and editing (equal). **Pierre Taberlet:** Investigation (equal); supervision (supporting); writing – review and editing (equal). **Erica van de Waal:** Conceptualization (equal); data curation (equal); funding acquisition (equal); project administration (equal); resources (equal); supervision (equal); validation (equal); writing – review and editing (equal). **Luca Fumagalli:** Conceptualization (equal); data curation (equal); funding acquisition (equal); project administration (equal); resources (equal); supervision (equal); validation (equal); writing – review and editing (equal).

## CONFLICT OF INTEREST

The authors note that PT is co‐inventor of a patent related to the Sper01 primers and the use of the P6 loop of the chloroplast trn*L* (UAA) intron for plant identification using degraded template DNA. This patent only restricts commercial applications and has no impact on the use of this locus by academic researchers.

## Supporting information


Appendix S1

## Data Availability

The DNA metabarcoding data generated for this study are available on DRYAD (10.5061/dryad.6q573n621). Sanger sequences for the local database have been deposited in GenBank under accession numbers OL898555‐OL898608.
