# Supplementary material for: Focal vs. fecal: Seasonal variation in the diet of wild vervet monkeys from observational and DNA metabarcoding data
Source: Ecol Evol. 2022 Oct 1;12(10):e9358. doi: 10.1002/ece3.9358 (PMC9526031; doi:10.1002/ece3.9358)
Supplement: Supplementary file 1 — Appendix S1 [file ECE3-12-e9358-s001.docx]

# Appendix S1

# Focal vs. faecal: seasonal variation in the diet of wild vervet monkeys from observational and DNA metabarcoding data

Loïc Brun, Judith Schneider, Eduard Mas Carrió, Pooja Dongre, Pierre Taberlet, Erica van de Waal and Luca Fumagalli

*Ecology and Evolution*

**Appendix S1: Table S1**: Species of local database, in bold species with identical sequences.

| Family | Species |
| --- | --- |
| Anacardiaceae | ***Searsia pyroides, Searsia natalensis*** |
| Anacardiaceae | *Sclerocarya birrea* |
| Araliaceae | *Cussonia spicata* |
| Arecaceae | *Phoenix reclinata* |
| Bignoniaceae | *Jacaranda mimosifolia* |
| Boraginaceae | *Ehretia rigida* |
| Burseraceae | *Commiphora neglecta* |
| Cactaceae | *Cereus jamacaru* |
| Cactaceae | *Opuntia ficus-indica* |
| Caesalpiniaceae | *Schotia brachypetala* |
| Capparaceae | *Boscia albitrunca* |
| Celastraceae | *Gymnosporia senegalensis* |
| Celtidaceae | *Celtis africana* |
| Combretaceae | *Combretum erythrophyllum* |
| Combretaceae | *Combretum apiculatum* |
| Ebenaceae | ***Euclea crispa, Euclea undulata, Diospyros dichrophylla*** |
| Euphorbiaceae | *Euphorbia ingens* |
| Euphorbiaceae | *Euphorbia tirucalli* |
| Fabaceae | *Dalbergia armata* |
| Fabaceae | *Dalbergia obovata* |
| Fabaceae | *Senna didymobotrya* |
| Fabaceae | *Sesbania punicea* |
| Fabaceae | *Mundulea sericea* |
| Flacourtiaceae | *Dovyalis caffra* |
| Lamiaceae | *Premna mooiensis* |
| Lamiaceae | *Volkameria glabra* |
| Meliaceae | *Melia azedarach* |
| Mimosaceae | ***Vachellia tortilis, Vachellia sieberiana*** |
| Mimosaceae | *Dichrostachys cinerea* |
| Mimosaceae, Caesalpiniaceae | ***Vachellia nilotica, Caesalpinia decapetala*** |
| Moraceae | *Ficus sycomorus* |
| Myrtaceae | *Psidium guajava* |
| Myrtaceae | *Eucalyptus camaldulensis* |
| Olacaceae | *Ximenia caffra* |
| Oleaceae | *Olea europaea* |
| Pentapetaceae | *Dombeya rotundifolia* |
| Rhamnaceae | *Berchemia zeyheri* |
| Rhamnaceae | *Ziziphus mucronata* |
| Rubiaceae | *Gardenia volkensii* |
| Rubiaceae | *Coddia rudis* |
| Rutaceae | *Citrus limon* |
| Sapindaceae | *Hippobromus pauciflorus* |
| Sapindaceae | *Pappea capensis* |
| Sapotaceae | *Sideroxylon inerme* |
| Solanaceae | *Solanum seaforthianum* |
| Solanaceae | *Solanum aculeastrum* |
| Verbenaceae | ***Lantana camara, Lippia javanica*** |
| Vitaceae | *Rhoicissus tridentata* |

**Appendix S1: Table S2** Species included in positive controls for Sper01 and Arth02 assays, in the order of 2-fold dilutions.

| Metabarcode | Species |
| --- | --- |
| Sper01 | *Taxus baccata, Salvia pratensis, Populus tremula, Rumex acetosa, Carpinus betulus, Fraxinus excelsior, Picea abies, Lonicera xylosteum, Abies alba, Acer campestre, Briza media, Rosa canina, Capsella bursa-pastoris, Geranium robertianum, Rhododendron ferrugineum, Lotus corniculatus* |
| Arth02 | *Acheta domesticus, Timema douglasi, Harmonia axyridis, Galleria mellonella, Pyrrhocoris apterus, Blaptica dubia, Isoperla rivulorum, Silo pallipes* |

**Appendix S1: Table S3** Plant genus and species in observational focal follows and detected in faecal samples. Plant indicators for seasons were identified using *Indicator value analyses* (Indval; Dufrêne & Legendre, 1997). For database assignments: L = assigned with local database, G = assigned with global database, NA = no sequence available for the used metabarcode.

| **Observational Data** | | | **DNA metabarcoding data** | | | | | **Plant category** |
| --- | --- | --- | --- | --- | --- | --- | --- | --- |
| **Genus/species observed** | **Frequency/12315** | **Season indicator  > 0.2, ***** | **Genus/species detected** | **Frequency/823** | **Total read counts** | **Season indicator**  **RRA > 0.2, ***** | **Data- base** |  |
| *Berchemia zeyheri* | 704 | summer+autumn,  0.397 | *Berchemia zeyheri* | 811 | 1285128 | summer+autumn,  0.460 | L | tree |
| *Boscia albitrunca* | 2 |  | *Boscia albitrunca* | 15 | 417 |  | L | tree |
| *Caesalpinia  decapetala* | 84 | spring, 0.255 | *V. nilotica/C. decapetala* | 82 | 4621 |  | L | tree/shrub |
| *Cereus jamacaru* | 752 | spring, 0.324 | *Cereus jamacaru* | 365 | 53092 |  | L | cactus |
| *Clausena anisata* | 2 |  | *Clausena anisata* | 0 | 0 |  | G | shrub |
| *Coddia rudis* | 145 | autumn, 0.236 | *Coddia rudis* | 31 | 324 |  | L | shrub |
| *Cussonia spicata* | 2 |  | *Cussonia spicata* | 0 | 0 |  | L | tree |
| *Dalbergia armata* | 92 |  | *Dalbergia armata* | 80 | 8113 | autumn+winter,  0.235 | L | liane |
| *Dichrostachys cinerea* | 1346 |  | *Dichrostachys cinerea* | 685 | 95218 | winter, 0.295 | L | small tree/shrub |
| *Dovyalis caffra* | 132 | spring, 0.340 | *Dovyalis caffra* | 559 | 163527 |  | L | small tree/shrub |
| *Ehretia rigida* | 785 | spring, 0.233 | *Ehretia rigida* | 523 | 236935 | spring, 0.585 | L | small tree/shrub |
| *Euclea crispa* | 249 | spring, 0.332 | *E. crispa/E. undulata/ D. dichrophylla* | 321 | 98890 | spring, 0.446 | L | tree |
| *Euclea undulata* | 16 |  | *E. crispa/E. undulata/ D. dichrophylla* | 321 | 98890 | spring, 0.446 | L | small tree/shrub |
| *Euphorbia ingens* | 6 |  | *Euphorbia ingens* | 0 | 0 |  | L | cactus |
| *Euphorbia tirucalli* | 3 |  | *Euphorbia tirucalli* | 0 | 0 |  | L | shrub |
| *Gardenia volkensii* | 51 |  | *Gardenia volkensii* | 23 | 483 |  | L | small tree/shrub |
| *Gymnosporia  senegalensis* | 29 |  | *Gymnosporia senegalensis* | 0 | 0 |  | L | small tree/shrub |
| *Hippobromus  pauciflorus* | 68 | summer, 0.244 | *Hippobromus pauciflorus* | 444 | 225809 | spring, 0.345 | L | tree |
| *Lantana camara* | 15 | autumn, 0.216 | *L. camara/Lippia javanica* | 202 | 14426 | autumn, 0.238 | L | shrub |
| *Opuntia ficus-indica* | 37 |  | *Opuntia ficus-indica* | 3 | 34 |  | L | cactus |
| *Premna mooiensis* | 391 | spring+summer,  0.276 | *Premna mooiensis* | 309 | 48541 | spring+summer,  0.223 | L | tree |
| *Schotia brachypetala* | 15 |  | *Schotia brachypetala* | 18 | 2490 |  | L | tree |
| *Searsia sp* | 688 | autumn+spring,  0.278 | *S. pyroides/S. natalensis* | 638 | 256910 | spring, 0.339 | L | tree/shrub |
| *Vachellia karroo* | 7 |  | *NA* | 0 | 0 |  | NA | tree |
| *Vachellia nilotica* | 1763 | autumn+winter,  0.400 | *V. nilotica/C. decapetala* | 82 | 4621 |  | L | tree/shrub |
| *Vachellia sp* | 323 | spring, 0.464 | *NA* | 0 | 0 |  | NA | tree |
| *Vachellia tortilis* | 2109 | autumn, 0.423 | *V. tortilis/V. sieberiana* | 817 | 772456 | autumn, 0.768 | L | tree |
| *Zanthoxylum capense* | 2 |  | *NA* | 0 | 0 |  | NA | tree |
| *Ziziphus mucronata* | 2497 | autumn+winter,  0.490 | *Ziziphus mucronata* | 765 | 511273 | winter, 0.681 | L | tree |
| *NA* | 0 |  | *Aizoon* | 467 | 50557 | autumn+winter,  0.203 | G | herb/shrub |
| *NA* | 0 |  | *Aloe* | 3 | 37 |  | G | various |
| *NA* | 0 |  | *Alternanthera pungens* | 4 | 208 |  | G | herb/shrub |
| *NA* | 0 |  | *Asparagus* | 309 | 18695 |  | G | herb/shrub |
| *NA* | 0 |  | *Basella alba* | 4 | 26 |  | G | climbing plant |
| *NA* | 0 |  | *Blepharis* | 3 | 41 |  | G | herb/shrub |
| *NA* | 0 |  | *Blepharis maderaspatensis* | 447 | 87987 |  | G | herb |
| *NA* | 0 |  | *Capparis* | 45 | 7161 |  | G | herb/shrub |
| *NA* | 0 |  | *Combretum* | 9 | 190 |  | G | shrub |
| *NA* | 0 |  | *Commiphora neglecta* | 2 | 17 |  | G | small tree/shrub |
| *NA* | 0 |  | *Crotalaria* | 205 | 38455 |  | G | herb/shrub |
| *NA* | 0 |  | *Diospyros* | 4 | 30 |  | G | tree |
| *NA* | 0 |  | *Dombeya rotundifolia* | 31 | 2112 |  | L | tree |
| *NA* | 0 |  | *Dysphania* | 5 | 65 |  | G | herb |
| *NA* | 0 |  | *Eragrostis superba* | 2 | 51 |  | G | grass |
| *NA* | 0 |  | *Erythrina* | 2 | 104 |  | G | tree |
| *NA* | 0 |  | *Euphorbia* | 9 | 343 |  | L | various |
| *NA* | 0 |  | *Ficus sycomorus* | 21 | 6481 |  | L | tree |
| *NA* | 0 |  | *Hibiscus* | 4 | 197 |  | G | shrub |
| *NA* | 0 |  | *Jasminum* | 699 | 114664 | autumn, 0.452 | G | shrub |
| *NA* | 0 |  | *Kohautia* | 2 | 16 |  | G | herb/shrub |
| *NA* | 0 |  | *Mundulea sericea* | 43 | 12193 |  | L | shrub |
| *NA* | 0 |  | *Ocimum* | 2 | 128 |  | G | herb/shrub |
| *NA* | 0 |  | *Ormosia* | 2 | 11 |  | G | small tree/shrub |
| *NA* | 0 |  | *Oxalis* | 325 | 36621 | spring, 0.218 | G | herb/shrub |
| *NA* | 0 |  | *Pappea capensis* | 6 | 144 |  | L | tree |
| *NA* | 0 |  | *Pereskia* | 4 | 24 |  | G | cactus |
| *NA* | 0 |  | *Phyllanthus maderaspatensis* | 19 | 2943 |  | G | herb |
| *NA* | 0 |  | *Plinthus* | 17 | 141 |  | G | herb/shrub |
| *NA* | 0 |  | *Priva* | 7 | 109 |  | G | herb/shrub |
| *NA* | 0 |  | *Priva cordifolia* | 555 | 69468 | winter+spring,  0.245 | G | herb/shrub |
| *NA* | 0 |  | *Psidium guajava* | 5 | 272 |  | L | shrub |
| *NA* | 0 |  | *Rhoicissus tridentata* | 212 | 31942 |  | L | shrub |
| *NA* | 0 |  | *Sclerocarya birrea* | 7 | 586 |  | L | tree |
| *NA* | 0 |  | *Senna didymobotrya* | 225 | 4949 | autumn+winter,  0.324 | L | herb/shrub |
| *NA* | 0 |  | *Sida* | 6 | 1040 |  | G | herb/shrub |
| *NA* | 0 |  | *Sideroxylon inerme* | 11 | 1098 |  | L | tree |
| *NA* | 0 |  | *Solanum seaforthianum* | 5 | 696 |  | L | shrub |
| *NA* | 0 |  | *Solidago virgaurea* | 3 | 524 |  | G | herb/shrub |
| *NA* | 0 |  | *Viscum minimum* | 37 | 6829 | autumn, 0.242 | G | hemi-parasite |
| *NA* | 0 |  | *Volkameria glabra* | 7 | 431 |  | L | tree |
| *NA* | 0 |  | *Vigna* | 2 | 47 |  | G | herb/shrub |
| *NA* | 0 |  | *Waltheria indica* | 6 | 217 |  | G | herb/shrub |

**Appendix S1: Table S4** Taxonomy of detected arthropod items in 823 faecal samples, total read counts over all samples.

| **Order** | **Family** | **Count** | **Frequency** |
| --- | --- | --- | --- |
| Araneae | Miturgidae | 3734 | 7 |
| Blattodea | Hodotermitidae | 98835 | 438 |
|  | Termitidae | 21619 | 46 |
| Coleoptera | NA | 59596 | 265 |
|  | Anthribidae | 297 | 3 |
|  | Brentidae | 12321 | 77 |
|  | Buprestidae | 295 | 4 |
|  | Chrysomelidae | 38345 | 300 |
|  | Curculionidae | 8126 | 18 |
|  | Elateridae | 321 | 2 |
|  | Hydrophilidae | 212 | 3 |
|  | Scarabaeidae | 572 | 6 |
|  | Tenebrionidae | 1076 | 8 |
| Diptera | NA | 3069 | 7 |
|  | Cecidomyiidae | 408 | 6 |
|  | Drosophilidae | 2702 | 20 |
|  | Tephritidae | 1645 | 9 |
| Hemiptera | Alydidae | 9594 | 131 |
|  | Cicadidae | 1286 | 2 |
|  | Coreidae | 250 | 7 |
|  | Pentatomidae | 1012 | 26 |
|  | Pyrrhocoridae | 197 | 7 |
| Lepidoptera | NA | 71362 | 372 |
|  | Erebidae | 464 | 5 |
|  | Lasiocampidae | 458 | 6 |
|  | Nepticulidae | 1446 | 24 |
|  | Noctuidae | 4724 | 52 |
|  | Nymphalidae | 248 | 5 |
| Mantodea | NA | 178 | 2 |
|  | Mantidae | 927 | 19 |
|  | Sibyllidae | 69 | 2 |
| Neuroptera | NA | 180 | 5 |
| Odonata | NA | 283 | 2 |
|  | Aeshnidae | 247 | 2 |
|  | Libellulidae | 149 | 2 |
| Orthoptera | NA | 611 | 6 |
|  | Acrididae | 7196 | 81 |
|  | Gryllacrididae | 170 | 4 |
|  | Gryllidae | 3636 | 29 |
|  | Pamphagidae | 783 | 11 |
|  | Tettigoniidae | 1368 | 19 |
| Thysanoptera | Thripidae | 29 | 2 |


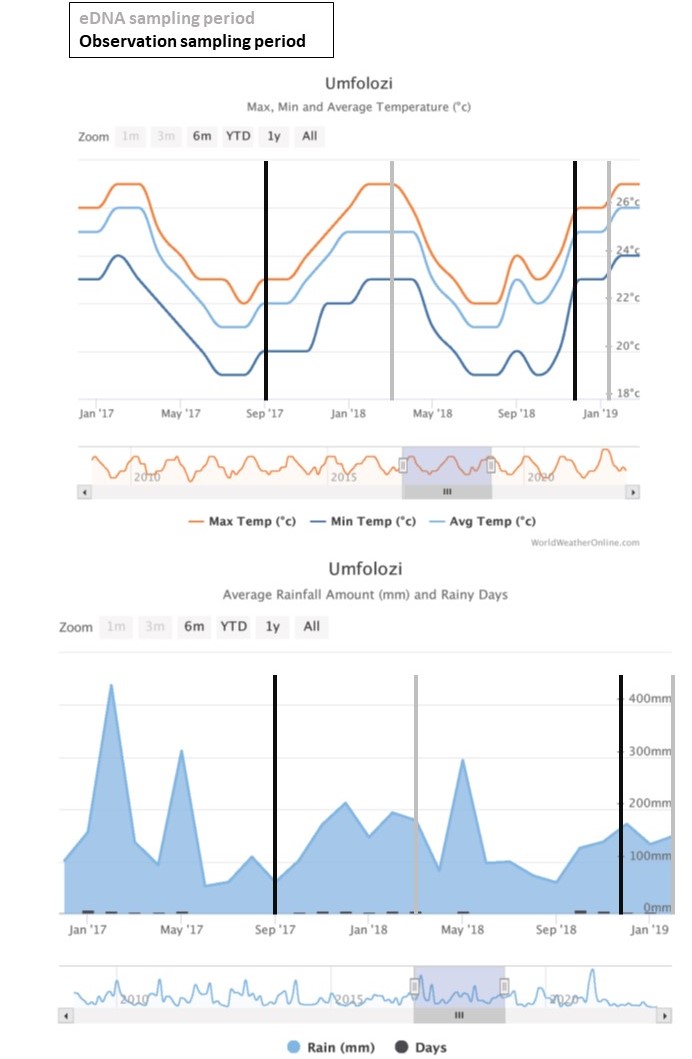


**Appendix S1: Figure S1**: Meteorological conditions have been assessed for the entire sampling period in terms of temperature (at the top; <https://www.worldweatheronline.com/umfolozi-weather-averages/kwazulu-natal/za.aspx>) and rainfall (at the bottom; https://www.weathersa.co.za/home/historicalrain). The period when focal screenings have been conducted is indicated in red, the one for eDNA sampling in green.


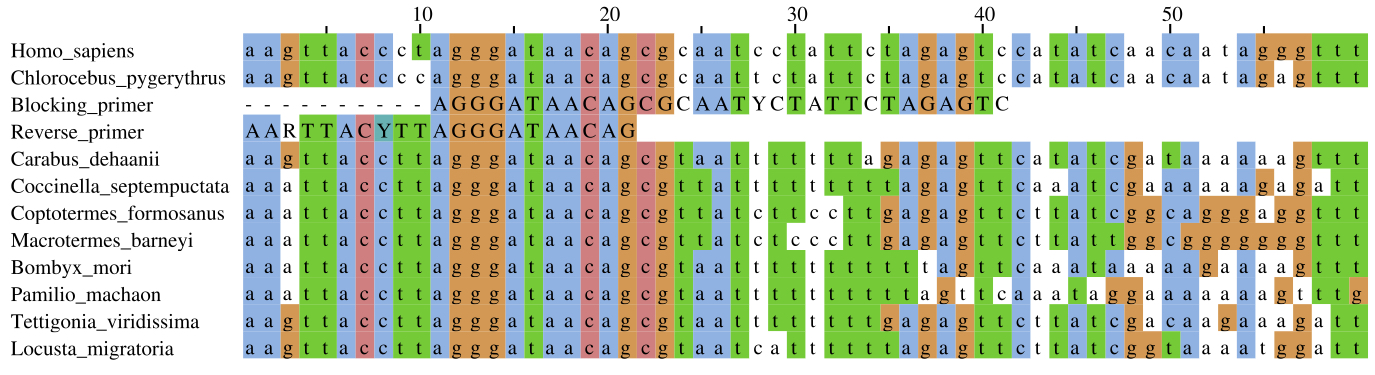


**Appendix S1: Figure S2.** Design of a blocking primer for vervet and human DNA for the Arth02 primer pair, allowing the amplification of target arthropod species. In this case, the 5'end of the blocking primer overlaps eleven nucleotides of the 3'-end of the reverse Arth02 amplification primer (Reverse_primer). The ideal position for the blocking primer is at the end of the PCR fragment where the variation between the sequence of the species to be blocked and the sequences of the target species is highest (Taberlet, Bonin, Zinger, & Coissac, 2018). A C3 carbon spacer must be added on the 3'-end of the blocking primer to prevent its 3'-extension (Vestheim & Jarman, 2008). In addition of human, vervet, blocking primer and Arth02 reverse primer, representative arthropod sequences are also shown in the alignment.

**Appendix S1: Figure S3.** Stacked bar plots resuming the proportion of read counts assigned to different taxonomic levels by using particular database options for **A.** the Sper01 assay and **B.** the Arth02 assay.


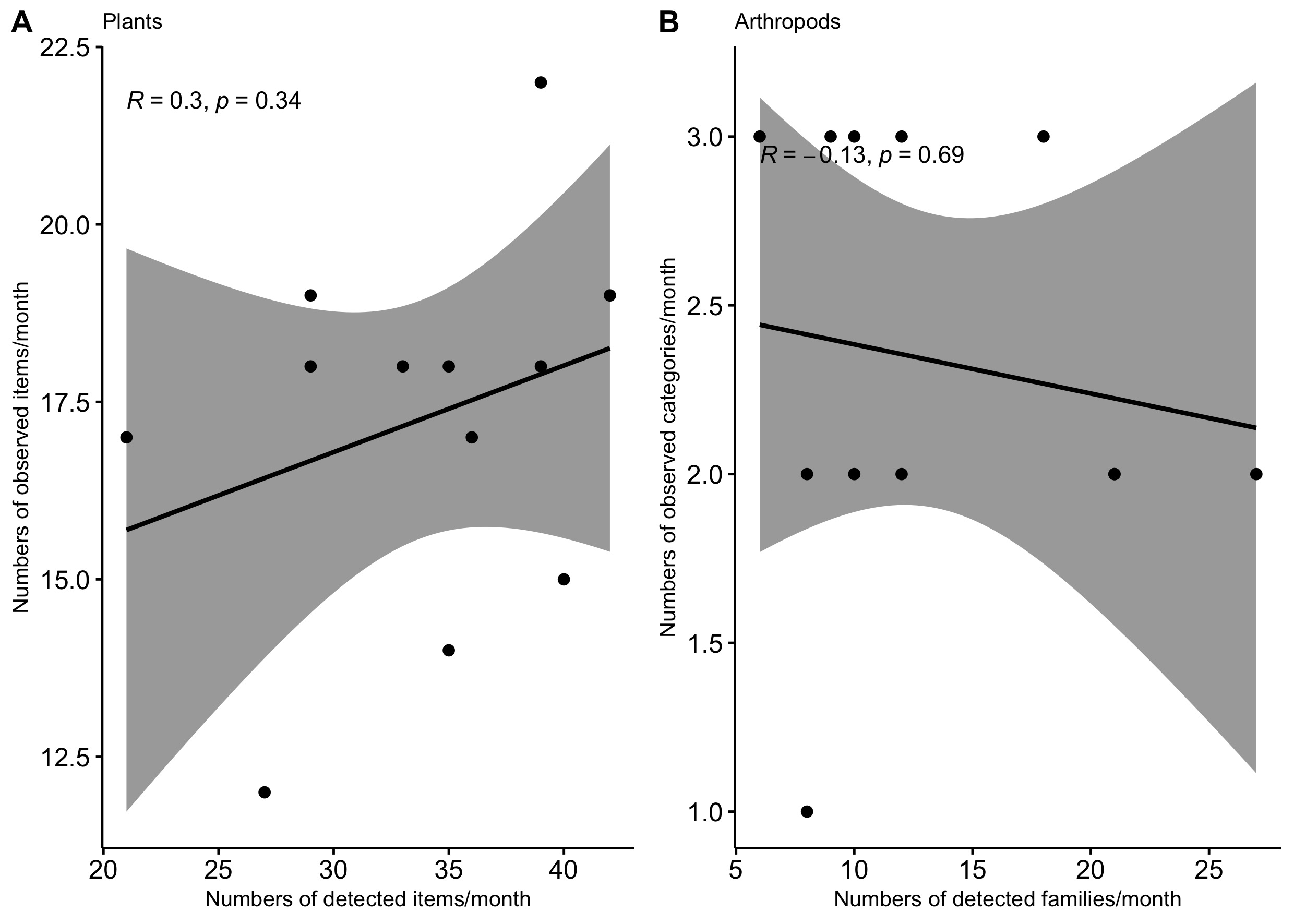


**Appendix S1: Figure S4**: Spearman rank correlations and coefficients based on total numbers per month of different dietary items as observed during focal follows and detected in faecal samples for **A.** plant data and **B.** arthropod data.


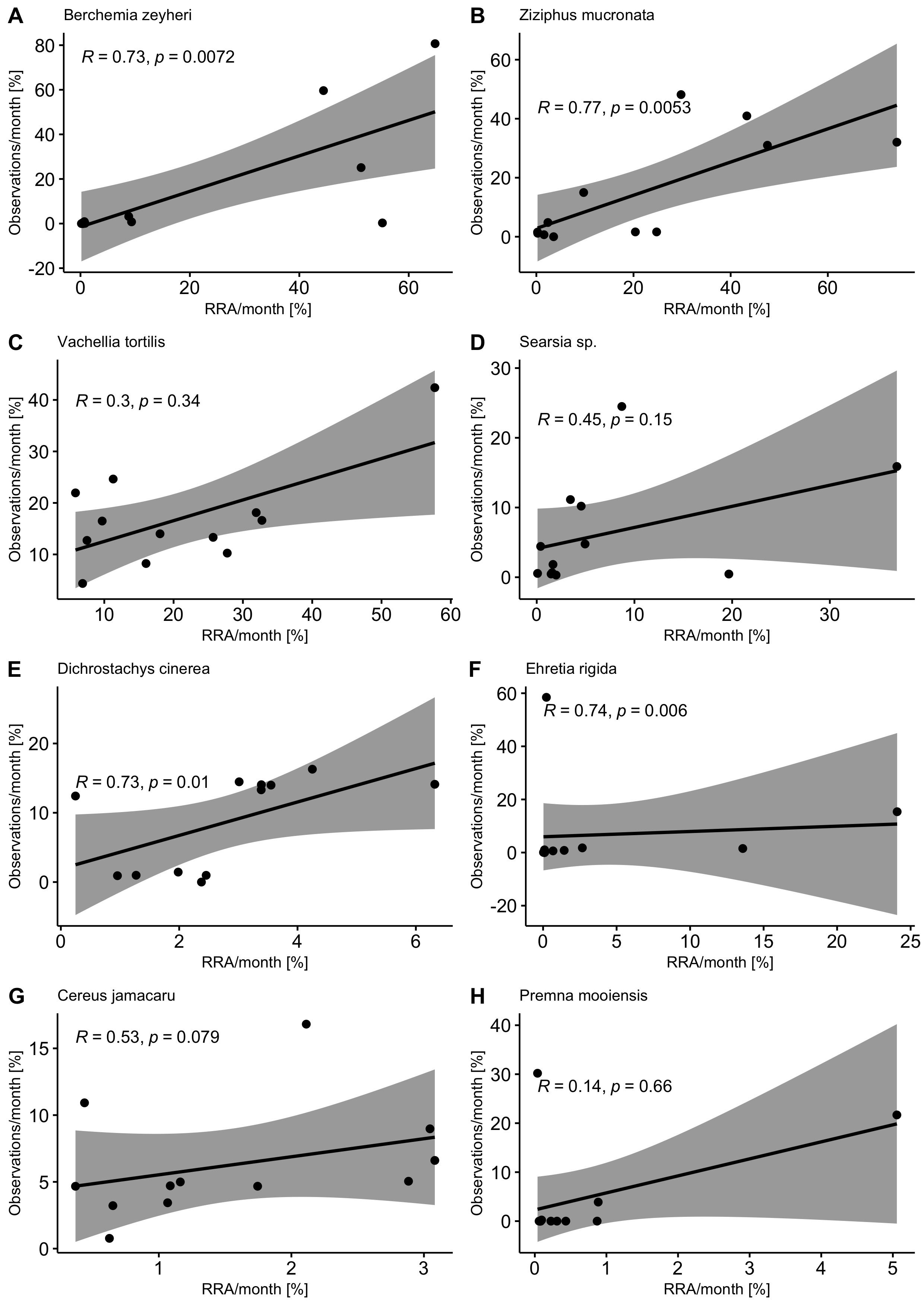


**Appendix S1: Figure S5**: Spearman rank correlations and coefficients based on mean count and RRA per month for all plant species present in both datasets and with a minimum of 350 observations, with the exception of those that had identical metabarcodes and matched several species in the focal dataset (i.e. *E. crispa*/*E. undulata*/*D. dichrophylla* and *V. nilotica*/*C. decapetala*). The observed plant *V. tortilis* corresponds to *V. tortilis/sieberiana* in the eDNA dataset.

**Appendix S1: Figure S6. A.** Mean counts per date of seven consumed plant species observed during focal screenings that are indicators for seasons (shown are those plants with Indval value > 0.2, which were observed > 350 times and which do not share sequences with other observed species, Appendix S1: Table S3). **B.** Mean RRA per date of the same seven consumed plant species in faecal samples, only included RRA > 0.001. All species, except *C. jamacaru*, are also season indicator species (> 0.2) in the RRA dataset (Appendix S1: Table S3). The observed plant *V. tortilis* corresponds to *V. tortilis/sieberiana* in the eDNA dataset. Note that this representation serves to compare methods and that there are a number of additional indicator species and genus in the metabarcoding dataset not included here (Appendix S1: Table S3).

**Appendix S1: Figure S7**: Spearman rank correlations and coefficients based on mean count and RRA per month for all arthropod categories as shown in Fig. 2B.

**Appendix S1: Figure S8**: Principal component analysis (PCA), based on relative read abundances (RRA) of consumed arthropod families detected in faecal samples (R2 = 3.6 %). The four seasons are represented by different colours and the texts represent the centroids.

**References**

Dufrêne, M., & Legendre, P. (1997). Species assemblages and indicator species: The need for a flexible asymmetrical approach. *Ecological Monographs*, *67*, 345–366. https://doi.org/10.2307/2963459

Taberlet, P., Bonin, A., Zinger, L., & Coissac, É. (2018). *Environmental DNA: For Biodiversity Research and Monitoring*. Oxford University Press. https://doi.org/10.1093/oso/9780198767220.001.0001

Vestheim, H., & Jarman, S. N. (2008). Blocking primers to enhance PCR amplification of rare sequences in mixed samples - A case study on prey DNA in Antarctic krill stomachs. *Frontiers in Zoology*, *5*, 1–11. https://doi.org/10.1186/1742-9994-5-12
